# Microbial communities of poultry house dust, excreta and litter are partially representative of microbiota of chicken caecum and ileum

**DOI:** 10.1371/journal.pone.0255633

**Published:** 2021-08-05

**Authors:** Yugal R. Bindari, Robert J. Moore, Thi Thu Hao Van, Matthew Hilliar, Shu-Biao Wu, Stephen W. Walkden-Brown, Priscilla F. Gerber

**Affiliations:** 1 Animal Science, School of Environmental and Rural Science, University of New England, Armidale, New South Wales, Australia; 2 School of Science, RMIT University, Bundoora West Campus, Bundoora, Victoria, Australia; USDA-Agricultural Research Service, UNITED STATES

## Abstract

Traditional sampling methods for the study of poultry gut microbiota preclude longitudinal studies as they require euthanasia of birds for the collection of caecal and ileal contents. Some recent research has investigated alternative sampling methods to overcome this issue. The main goal of this study was to assess to what extent the microbial composition of non-invasive samples (excreta, litter and poultry dust) are representative of invasive samples (caecal and ileal contents). The microbiota of excreta, dust, litter, caecal and ileal contents (n = 110) was assessed using 16S ribosomal RNA gene amplicon sequencing. Of the operational taxonomic units (OTUs) detected in caecal contents, 99.7% were also detected in dust, 98.6% in litter and 100% in excreta. Of the OTUs detected in ileal contents, 99.8% were detected in dust, 99.3% in litter and 95.3% in excreta. Although the majority of the OTUs found in invasive samples were detected in non-invasive samples, the relative abundance of members of the microbial communities of these groups were different, as shown by beta diversity measures. Under the conditions of this study, correlation analysis showed that dust could be used as a proxy for ileal and caecal contents to detect the abundance of the phylum *Firmicutes*, and excreta as a proxy of caecal contents for the detection of *Tenericutes*. Similarly, litter could be used as a proxy for caecal contents to detect the abundance of *Firmicutes* and *Tenericutes*. However, none of the non-invasive samples could be used to infer the overall abundance of OTUs observed in invasive samples. In conclusion, non-invasive samples could be used to detect the presence and absence of the majority of the OTUs found in invasive samples, but could not accurately reflect the microbial community structure of invasive samples.

## Introduction

The large and complex communities of bacteria that inhabit the mucosa and lumen of the intestinal tract play an important role in host health, nutrition and physiology [1, 2]. Caecal microbiota has been widely investigated in birds because of its role in fermentation and digestion of complex carbohydrates such as cellulose, starch, and resistant polysaccharides. This digestion results in the production of short-chain fatty acids which influence bird health and performance [3–5]. Caeca also play a major role in water absorption [6–8]. The ileum is important for nutrient digestion and absorption but its microbiota is less diverse [9, 10]. Caecal microbiota is qualitatively and quantitatively the most diverse and functionally the most complex in birds compared to other parts of the intestinal tract [11]. Sampling of caecal contents or other sections of the intestinal tract is labour-intensive and requires the bird to be sacrificed which may add variability in studies due to animal to animal microbiota variation [4]. Additionally, longitudinal studies are not possible when using invasive sampling as samples can only be collected at a single time-point from each bird. Thus, some research effort has been devoted to identifying and characterising non-invasive samples as an alternative to intestinal samples. This would contribute both to reduce the noise in the data, as the same birds would be sampled, and decrease the number of birds required [12], which is in line with the current framework for humane animal research.

The microbiota found in commonly used non-invasive samples, such as cloacal swabs and excreta are not stable and abrupt temporal fluctuations occur due to the cyclic emptying of the different regions of the gastrointestinal tract [13]. Overall, *Firmicutes* is the most dominant phylum in the chicken intestine [14]. *Lactobacillus* is the primary bacterial taxa found in the crop, gizzard, duodenum and ileum while *Bifidobacterium* and *Enterobacter* are also commonly detected in the crop [15, 16]. *Clostridium*, *Ruminococcus*, *Streptococcus*, *Candidatus*, *Arthromitus* (phylum *Firmicutes*) and *Escherichia* and *Enterococcus* (phylum *Proteobacteria*) have also been reported in the ileum [16–20]. The most abundant phyla found in the caecum are *Firmicutes* followed by *Bacteroidetes* while *Proteobacteria* and *Archaea* are present in lesser amounts [21–24]. The most dominant bacterial taxa in the caecum within the phylum *Firmicutes* were found to be *Clostridiaceae*, *Lactobacillus*, *Ruminococcus*, *Faecalibacterium*, *Bacteroidaceae*, *Clostridium*, *Sporobacter*, *Oscillospira*, *Acetanaaerobacterium*, *Subdoligranulum* and *Pseudobutyrivibrio* [18, 19, 25, 26]. *Lactobacillus*, *Clostridium*, *Faecalibacterium*, *Ruminococcus*, *Bacillus*, *Eubacterium* (phylum *Firmicutes*) are predominant in cloaca and excreta [3, 27, 28]. Although excreta has been shown to contain microbial taxa present in the caecum, there are quantitative differences in the microbial composition between sample types [4].

Microbial communities of cloacal swabs have been shown to be representative of litter samples, and to a lesser extent representative of ileum samples, while cloacal swabs are not representative of caecal contents [4, 29]. No difference in bacterial genera has been reported between cloacal swabs and ileal contents at day 2, 7 and 14 while *Faecalibacterium*, *Blautia* and *Enterococcus* have been found to be highly abundant in excreta compared to ileal contents at day 35 [30]. Caecal droppings have been suggested as a useful alternative to study caecal microbiota composition longitudinally as no difference in bacterial genera has been reported between these sample types, except for a higher abundance of facultative anaerobic bacteria such as *Lactobacillus* in caecal droppings [12, 31]. The collection of caecal droppings is, however, more labour-intensive than cloacal swabs or excreta as chickens produce a caecal dropping after 7–8 excreta droppings [32].

Non-invasive population-level samples have also been explored for their ability to evaluate the gut microbiota [31]. The microbiota of boot sock samples, which are a mixture of excreta and litter, was reflective of caecal microbiota composition early in the grow-out period (days 0 to 14) but not at later stages [31]. The management, quality and type of litter, which is a mixture of a bedding material (e.g. wood shavings, straw) and bird excreta, have been demonstrated to directly influence the gut microbiota composition in a reciprocal manner [33]. *Lactobacillus* has been shown to be a predominant bacterial taxa in the ileal mucosa of chickens raised on fresh pine shavings while unclassified groups of *Clostridiales* were dominant in the ileal mucosa of chickens raised on reused litter [33].

Poultry house dust could potentially be used as a non-invasive sample type, at the population level, for monitoring flock microbiota. Poultry dust is a mixture of dried excreta, exfoliated skin and feathers, feed, litter and microorganisms that become aerosolised during human and chicken movement in the poultry house [34]. Excreta is the major component of poultry house dust in turkey [35] and meat chicken flocks [34, 36]. A recent study by Luiken et al. [37] that investigated antimicrobial resistant bacteria in farmers and livestock, showed that the microbiota of settled poultry house dust are associated with the microbiota of chicken excreta, providing evidence that this approach could be useful for the study of gut microbiota. In addition, previous studies on molecular testing of poultry dust for population-level monitoring of viruses, protozoa, and bacteria in commercial flocks have shown that dust samples collected at any location of the poultry house could be used to detect microorganisms of interest even at relative low levels of infection (approximately 20% of infected birds) [38–44], suggesting that this may be a practical sample type for population-level monitoring of pathogens and perhaps gut microbiota. This study aimed to compare non-invasive samples (dust, excreta, and litter) to invasive (caecal and ileal contents) sampling methods for studying microbiota composition. Samples were collected from *Eimeria* and *Clostridium perfringens* challenged and unchallenged birds; however, no difference in microbial communities between challenged and unchallenged birds was observed. Therefore, the data obtained from both groups were combined and analysed together to test the following propositions. 1) The comparison of the microbial communities of non-invasive samples (dust, excreta and litter) with invasive samples (ileal and caecal contents) will show similarities in the detected operational taxonomic units but quantitative differences in their abundance; and 2) the comparison of microbial communities of non-invasive samples will show similarities in the detected operational taxonomic units and their abundance.

## Methodology

### Sample collection

Samples used in this study were collected as part of a previous experiment [45] that was approved by the University of New England Animal Ethics Committee (AEC18-059). Briefly, 972 day-old Ross cockerels (Aviagen breeder hatchery, Goulburn, New South Wales, Australia) were assigned into a ‘challenged’ or an ‘unchallenged room’ containing 72 equal sized floor pens for rearing chickens. Birds were randomly allocated to a group of 13–14 birds into each of the floor pens (120 × 75 cm) covered with fresh wood shavings at approximately 7 cm depth on arrival [45]. The facility underwent a deep clean before placement of chickens, to minimise the microbial load from previous use. On day 9, birds in the challenged room were orally gavaged 2,500 sporulated oocysts of *Eimeria brunetti*, and 5,000 sporulated oocysts each of *E*. *maxima* and *E*. *acervulina* (Eimeria Pty Ltd., Ringwood, Victoria, Australia) in 1 mL phosphate buffer solution. Birds in the unchallenged room were given 1 mL sterile phosphate buffer solution. Challenged birds were orally gavaged with 1 ml of approximately 10^8^ colony forming units per ml of *C*. *perfringens* suspension in thioglycolate broth on day 14 with a repeat dose on day 15 for the induction of subclinical necrotic enteritis [45]. Unchallenged birds received 1 mL of sterile thioglycolate broth each day.

Birds were fed with starter diet (24.6% CP) until day 7, which was composed of wheat, sorghum, and soybean meal (3,000 kcal/kg). After day 7, birds were fed with pelleted diets isoenergetic at 3,080 kcal/kg for the grower diet (7–21 days) and 3,100 kcal/kg (days 21–35) for the finisher diet. Two pens in which chickens were fed with an industry-standard diet (S1 Table), were selected from both challenged and unchallenged rooms for sample collection in this study.

Excreta swab and litter samples from the selected pens were collected at days 0, 7, 13, 16, 21, 28 and 35. For the collection of fresh excreta, a clean white paper was placed on the floor of the selected pens and fresh excreta was immediately sampled by rolling a flocked swab (FLOQSwabs, Copan, Brescia, Italy) until all its surface was covered with excreta from one individual bird per pen, total of 28 samples. Two excreta samples were discarded due to poor quality DNA making a total of 26 excreta samples analysed in this study. Dust samples were collected using two dust plates (surface area of 520 cm^2^ each) suspended at approximately 1.5 m height in each room as previously described [46], total of 24 samples. Briefly, the dust plates were installed at chick placement and dust settled on the plates was scraped using a tissue into a ziplock bag at days 7, 13, 16, 21, 28 and 35. After each dust collection, the plates were repositioned until the next collection day. The settled dust was dry and there was enough accumulated material for testing (at least 50 mg dust) in each sampling day.

Swabs of ileal and caecal contents were collected from two birds per pen shortly after euthanasia on days 16 and 35, total of 32 samples, and were immediately placed on ice until arrival to the laboratory. Litter was collected from nine different locations per pen using a spatula and pooled together (approximately 4 g/pen) to form a single sample per pen, total of 28 samples. The litter samples were collected without entering the pen. Once transported to the laboratory, each pooled litter was dried in an oven at 36°C for up to 7 days and was ground, using an ultra-centrifugal grinder (ZM 200, Retsch, Germany), to pass through a 2 mm sieve, and thoroughly mixed. All samples were stored at -20°C until further analysis.

### DNA extraction and 16S rRNA sequencing

DNA extraction was performed using DNeasy PowerSoil Pro Kit (Qiagen, Hilden, Germany) according to the manufacturer’s instructions, with minor modifications. Briefly, all samples (swabs of excreta, ileal and caecal contents, 10 mg dust, 50 mg excreta or 50 mg litter) were added to powerbead pro tubes, resuspended in 800 μl of lysis buffer and homogenised for 5 min at maximum speed in a bullet blender (Model BBY24M-CE, Sigma Aldrich, St. Louis, USA) with subsequent heating of the homogenised suspension at 90°C for 10 min. A negative control (no sample added) was used in each DNA extraction batch. DNA concentration of each sample was measured using a Nanodrop before proceeding to the library generation step. Amplicons of the V3-V4 region of 16S rRNA genes, targeting the 343–806 bp region, were produced using forward (ACTCCTACGGGAGGCAGCAG) and reverse (GGACTACHVGGGTWTCTAAT) primers. The primers also contained barcodes, spacer sequences, and Illumina sequencing linkers [47]. Water was used as a negative control for each PCR amplification in a 96 well plate. The sequencing of the amplicons was performed on the Illumina MiSeq platform using 2 × 300 bp paired-end reads and sequence data were processed in QIIME [48]. Paired-end sequences were combined using Fastq-Join algorithm allowing no mismatch within overlapped regions. The reads with the Phred quality scores of 20 or above were used for analysis. The operational taxonomic unit (OTUs) were picked at 97% similarity using UCLUST [49]. Chimera sequences were checked using Pintail [50] and taxonomy assignment was performed against the GreenGenes database (v 2013_8) using QIIME default parameters [51]. After quality trimming, rare sequences that represented Operational Taxonomic Units (OTUs) present at less than 0.01% of the total sequences, were removed from further analysis. The data set consisted of 2,668,239 sequences, with an average of 24,257 sequences per sample. The sequence data is publicity available at the MG-RAST database under a project ID mgm4926609.3 and library ID mgl839711.

### Statistical analysis

Hellinger transformation was used for normalizing microbial community composition data before statistical analyses, which were performed using Calypso software [52]. Alpha diversity was calculated using the Shannon index, which is a measure of species richness and evenness, and Chao1, which also measures microbial richness but gives more weight to low abundance species compared to Shannon [53]. The difference in microbial community composition between the samples types were visualized using principal coordinate analysis (PCoA) using Bray–Curtis distance metric, as a quantitative measure of beta diversity (abundance of OTUs), and Jaccard distance metric, as a qualitative measure (presence/absence of OTUs) [54]. Hierarchical clustering was performed on Bray–Curtis and Jaccard distance metrics to visualize the cluster of different samples and the output was shown as dendrograms. Homogeneity of multivariate dispersion was tested using permutational analyses of multivariate dispersions (PERMDISP) on Bray–Curtis and Jaccard distance metrics [55]. The overall difference in microbial community between the sample types for the challenged and unchallenged groups was investigated using permutational multivariate analysis of variances (PERMANOVA) on Bray–Curtis and Jaccard distance metrics using the Adonis function. Venn diagrams were constructed to visualise common bacterial taxa and linear discriminant analysis effect size (LEfSe) was performed to find the most discriminatory features between the sample types. Spearman rho ranked order correlation coefficient was performed to investigate if non-invasive samples (dust, excreta and litter) could serve as a proxy to study the microbiota of invasive sampling methods (ileal and caecal contents). Correlation coefficient values < 0.1 were considered as negligible correlation, 0.10–0.39 as weak, 0.40–0.69 as moderate, 0.70–0.89 as strong and > 0.9 were considered very strong correlation [56]. Non-invasive samples (dust, excreta and litter) were considered as a proxy for invasive samples (caecal and ileal contents) if the correlation of the microbial abundance between non-invasive and invasive samples was strong (≥ 70).

## Results

There was no significant difference in the microbial communities between challenged and unchallenged birds for any sample type (S2 Table), therefore the data of both groups were combined and analysed together. Because of the low number of samples collected for each age group, samples collected after challenge with *Eimeria* spp. were combined. The results of the study are presented below.

### Microbial alpha diversity

**Fig 1:**
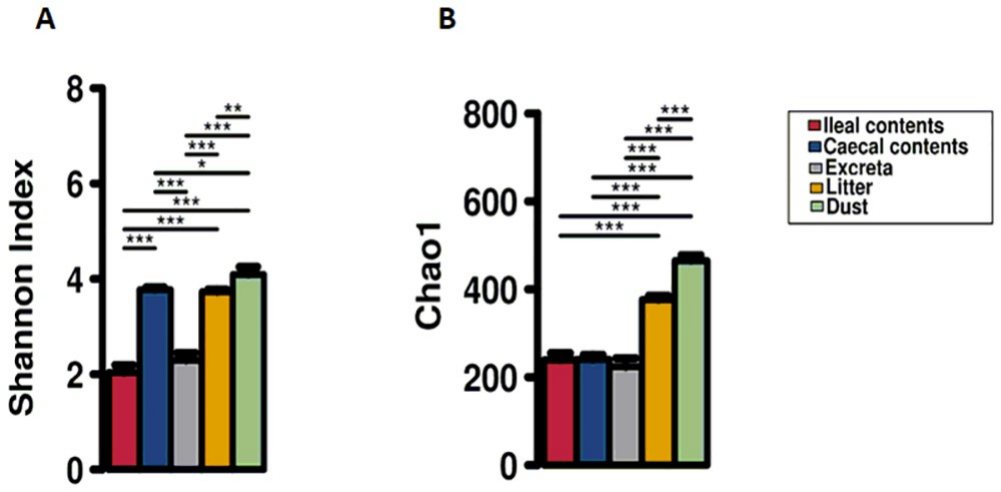
Alpha diversity assessed by Shannon diversity (A) and Chao1 richness (B). *Denotes significant difference at the P < 0.05 level, ** denotes significant difference at P < 0.01 level, *** denotes significant difference at the P < 0.001 level between sample types connected by a line.

Overall, the highest microbial diversity was observed in dust samples and the lowest was observed in excreta and ileal contents using both Shannon and Chao1 alpha-diversity indices (Fig 1). Shannon diversity was similar in caecal contents and litter (*P* = 0.21), and the diversity in those samples were higher than ileal contents and excreta (*P* < 0.001) (Fig 1). Chao1 diversity was higher in litter compared to excreta, ileal and caecal contents (*P* < 0.001) and was similar among the latter samples (Fig 1). The number of OTUs found in caecal, ileal and excreta was lesser than previous studies [57–59] because of the stricter quality control and trimming steps applied in this study for removing sequences of low quality and low abundance OTUs.

### Microbial community structure

**Fig 2:**
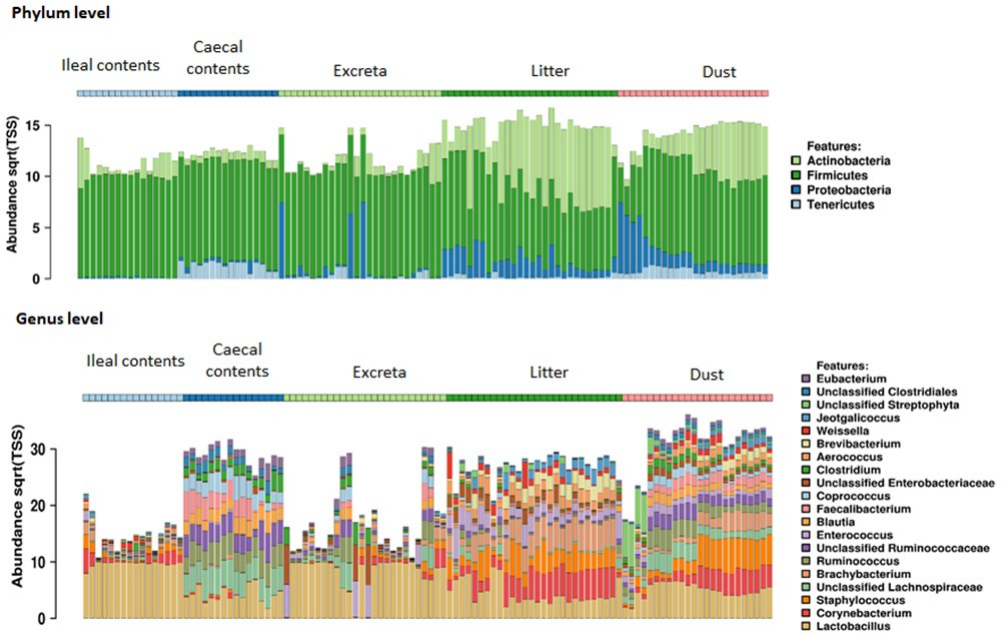
Abundance at the phylum level and top 20 most abundant genera for each sample type (caecal and ileal contents, dust, excreta and litter).

The microbial composition of ileal and caecal contents, excreta, litter, and dust samples at phylum and genus level are shown in Fig 2. There was a clear differentiation of the phylogenetic structure of each sample type at phylum and genus levels (Fig 2).

**Fig 3:**
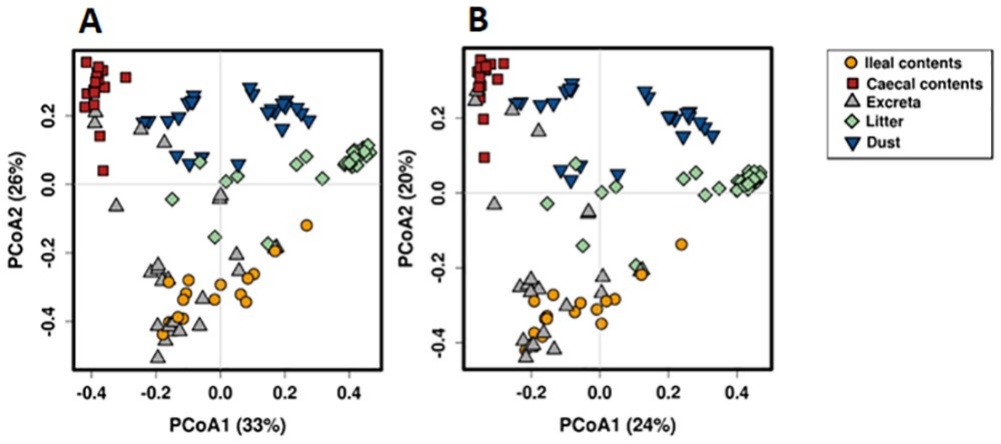
Principal coordinates analysis (PCoA) using Bray-Curtis (A) and Jaccard distance metric (B) showing the difference in the microbial community between caecal and ileal contents, dust, excreta and litter samples.

The clustering by sample type was also evident in the PCoA plots in which most samples tended to cluster together based on their source, with some interspersion between sample sources, particularly among ileal contents and excreta (Fig 3, S1 Fig). Microbiota of all sample types contained microbial taxa of phyla *Firmicutes*, *Actinobacteria*, *Proteobacteria* and *Tenericutes* (Fig 2), with *Firmicutes* the most abundant phyla in all sample types. *Lactobacillus* was the most dominant genera in ileal contents, dust, excreta and litter samples while unclassified *Lachnospraceae* was the most dominant genera in caecal contents (Fig 2).

Overall, the microbial communities of each sample type were significantly different from each other in both Adonis Bray-Curtis and Jaccard diversity measures on pair-wise comparisons (Table 1).

**Table 1:** Difference in the microbial community between the two groups measured using permutational multivariate analysis of variances (PERMANOVA) on Bray–Curtis and Jaccard distance metrics using the Adonis function.

Comparison	Adonis Bray-Curtis			Adonis Jaccard		
	R-square	P-value	PERMDISP (P-value)	R-square	P-value	PERMDISP (P-value)
Caecal contents vs dust	0.41	**<0.001**	0.54	0.32	**<0.001**	0.73
Caecal contents vs litter	0.61	**<0.001**	0.43	0.45	**<0.001**	0.56
Caecal vs ileal contents	0.58	**<0.001**	**0.005**	0.43	**<0.001**	**0.005**
Caecal contents vs excreta	0.32	**<0.001**	**<0.001**	0.25	**<0.001**	**<0.001**
Ileal contents vs dust	0.41	**<0.001**	0.09	0.31	**<0.001**	0.07
Ileal contents vs litter	0.38	**<0.001**	0.19	0.28	**<0.001**	0.18
Ileal contents vs excreta	0.06	**0.02**	0.06	0.05	**0.02**	**0.04**
Dust vs excreta	0.27	**<0.001**	**<0.001**	0.22	**<0.001**	**<0.001**
Dust vs litter	0.25	**<0.001**	0.81	0.21	**<0.001**	0.78
Litter vs excreta	0.31	**<0.001**	**0.001**	0.25	**<0.001**	0.73

PERMDISP = permutational multivariate dispersions test calculated using Bray-Curtis and Jaccard distance metrics. R-square is the proportion of the variance explained by the group. Bolded values signify statistical difference at a P<0.05 level.

**Fig 4:**
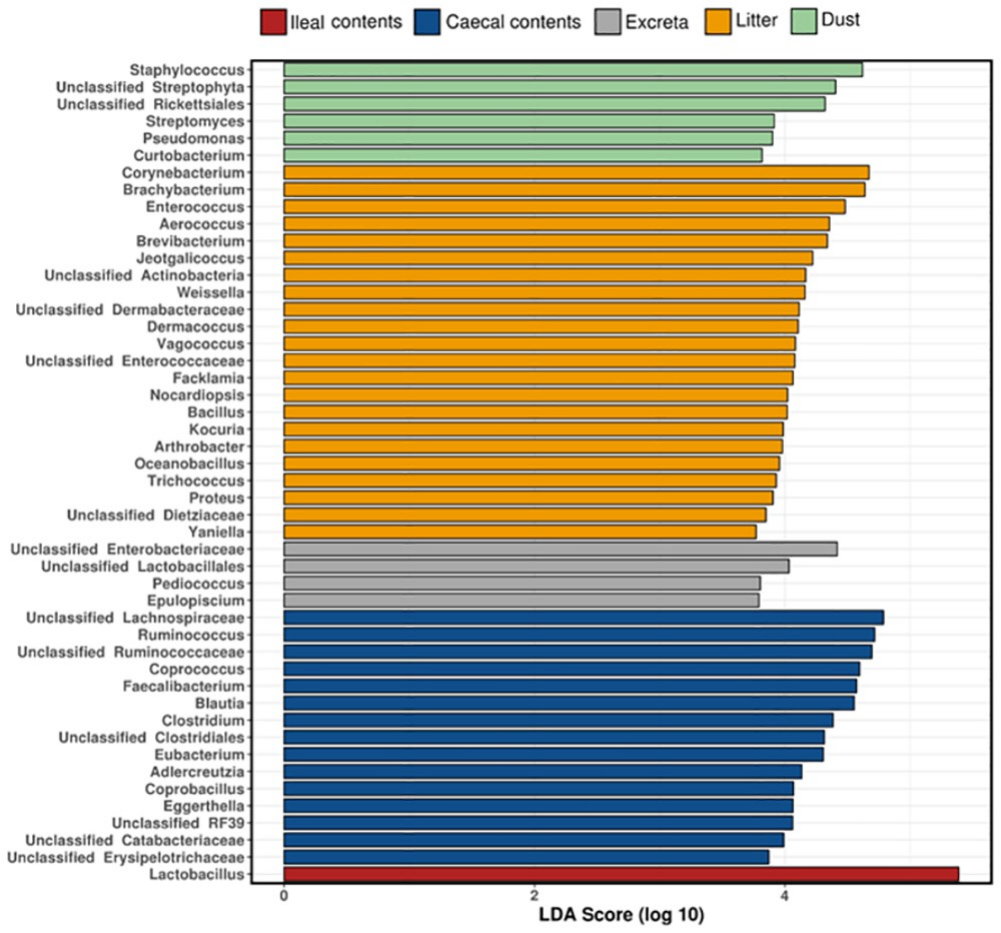
Genera that differentiate sample types identified using linear discriminant analysis effect size (LEfSe).

The elements of the microbiota that are most responsible for differentiating the composition of the different sample types are shown in Fig 4.

### The proportion of operational taxonomic units (OTUs) shared by invasive and non-invasive samples

**Fig 5:**
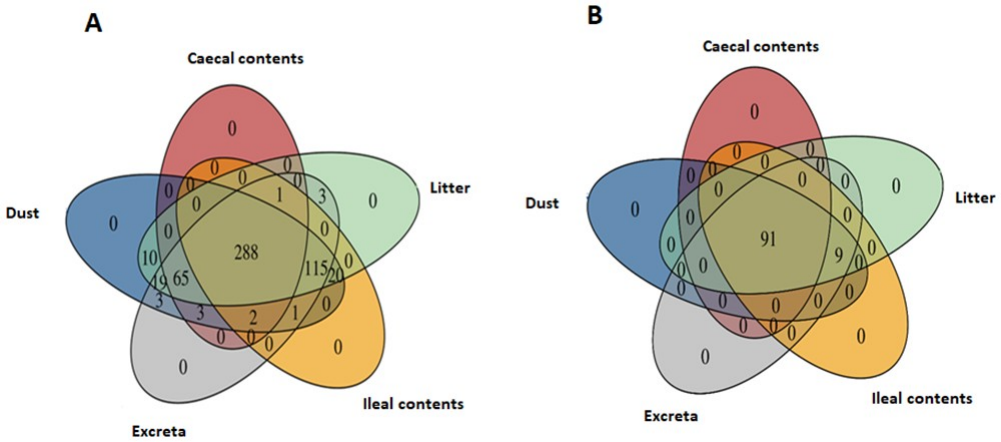
Venn diagram showing common bacterial OTUs between sample types A) across all OTUs and in the B) top 100 most abundant OTUs.

The number of OTUs in dust (n = 526), litter (n = 521) and excreta (n = 500) was higher than in ileal (n = 427) and caecal contents (n = 359) (Fig 5A, S3 Table). Although the number of OTUs were higher in non-invasive samples compared to invasive samples, most of the OTUs found in caecal and ileal contents were detected in dust, litter and excreta. A total of 99.7% of OTUs detected in caecal contents were also detected in dust, while 98.6% were detected in litter and 100% in excreta (Fig 5A, S4 Table). *Lactobacillus helveticus* (otu_256176) was detected in caecal contents but not in dust [abundance sqrt (TSS) = 0.01, occurrence = 12%]. *Lachnospiraceae* (otu_884077), *Catabacteriaceae* (otu_1033420), *RF39* (otu_764779), *Lachnospiraceae* (otu_351809) and *Blautia* (otu_961773) were detected in caecal contents but not in litter [abundance sqrt (TSS) = 0.24–0.40, occurrence = 38–94%].

A total of 99.8% of OTUs detected in ileal contents were also detected in dust, 99.3% in litter and 95.3% in excreta (Fig 5A). *Lactobacillus helveticus* (otu_256176) was detected in ileal contents but not in dust [abundance sqrt (TSS) = 0.1, occurrence = 38%]. Similarly, *Blautia* (otu_961773), *Lachnospiraceae* (otu_884077) and *Propionibacterium acnes* (otu_522210) were detected in ileal contents but not detected in litter [abundance sqrt (TSS) = 0.004, occurrence = 6%]. There were 20 OTUs detected in ileal contents but not in excreta (S5 Table); however, they were not part of the ileal core OTUs as their occurrence and abundance was relatively low [abundance sqrt (TSS) = 0.003–0.03, occurrence = 6–31%]. The OTUs found in dust, excreta and litter but absent in ileal or caecal contents are presented in S5 Table.

Dust, excreta and litter shared 92% of total detected OTUs among them. A total of 99.2% of OTUs detected in excreta and litter were also detected in dust (Fig 5A). The OTUs detected in excreta and litter but absent in dust were *Actinobacteria* (otu_216320), *Clostridium* (otu_306483), *Lactobacillus helveticus* (otu_256176) and *Enterococcus faecalis* (otu_115349) [abundance sqrt (TSS) = 0.003–0.12].

When the microbial composition between the sample types were compared across top 100 OTUs, 91% of the total detected OTUs were shared between dust, litter and caecal contents while 100% were shared between litter, dust and ileal contents (Fig 5B).

### Correlation of microbial composition between invasive samples and non-invasive samples

The overall microbial abundance of non-invasive samples (excreta, dust, litter) was weakly to moderately correlated (Spearman r_s_ < 70) with invasive samples (caecal and ileal contents) (S6 Table). Overall, there was a moderate positive correlation of OTUs abundance between caecal contents and dust (r_s_ = 0.41; *P* < 0.001), and caecal contents and excreta (r_s_ = 0.58; *P* < 0.001), while a moderate negative correlation was found between caecal contents and litter (r_s_ = -0.46; *P* < 0.001). Similarly, moderate positive correlation of the OTUs abundance was found between ileal contents and excreta (r_s_ = 0.49; *P* < 0.001), and ileal contents and litter (r_s_ = 0.55; *P* < 0.001), while a weak positive correlation was found between ileal contents and dust (r_s_ = 0.34; *P* < 0.001).

No phylum was significantly positively correlated in abundance between ileal contents and excreta (*P* > 0.05) (S7 Table). *Tenericutes* was the only phylum showing a strong positive correlation between caecal contents and excreta (r_s_ = 0.78, *P* < 0.001). Strong positive correlations between caecal contents and dust, and caecal contents and litter samples were found for the phylum *Firmicutes* (r_s_ = 0.83–0.85, *P* < 0.001) while negative correlations between caecal contents and dust, and caecal contents and litter were found for *Actinobacteria* and *Proteobacteria* (r_s_ = -0.71 –-0.82, *P* < 0.001). Strong positive correlation between caecal contents and litter was also found for the phylum *Tenericutes* (r_s_ = 0.81, *P* < 0.001) (S7 Table). Similarly, strong positive correlation between ileal contents and dust, and ileal contents and litter was found for *Firmicutes* (r_s_ = 0.75–0.77, *P* < 0.001) and negative correlation was found for *Tenericutes*, *Proteobacteria* and *Actinobacteria* (r_s_ = -0.50 –-0.85, *P* < 0.001). The correlation of the microbiota at the genus level between non-invasive samples and invasive samples are shown in Table 2.

**Table 2:** Spearman’s rank correlation between the relative abundance of genera of non-invasive samples (dust, excreta and litter) and invasive samples (ileal and caecal contents).

Genera	Spearman’s rank correlation					
	Caecal contents and litter	Caecal contents and dust	Ileal contents and dust	Ileal contents and litter	Caecal contents and excreta	Ileal contents and excreta
*Acinetobacter*	-0.80	-0.88	-0.85	-0.75	-0.12	0.25
*Adlercreutzia*	0.84	0.74	-0.83	-0.38	0.71	-0.31
*Aerococcus*	-0.84	-0.84	-0.68	-0.75	-0.28	0.28
*Arthrobacter*	-0.80	-0.88	-0.88	-0.8	-0.12	-0.12
*Bacillus*	-0.79	-0.67	-0.57	-0.76	0.05	0.37
*Blautia*	0.83	0.8	-0.85	-0.38	0.81	-0.18
*Brachybacterium*	-0.83	-0.8	-0.57	-0.76	-0.20	0.26
*Brevibacterium*	-0.84	-0.85	-0.63	-0.77	-0.25	0.27
*Burkholderia*	-0.12	-0.19	-0.05	0.06	NA	0.2
*Clostridium*	0.83	0.68	-0.77	-0.52	0.55	-0.48
*Coprobacillus*	0.8	0.4	-0.86	-0.39	0.78	-0.25
*Coprococcus*	0.83	0.81	-0.85	-0.37	0.73	-0.17
*Corynebacterium*	-0.83	-0.77	-0.42	-0.63	-0.18	0.36
*Curtobacterium*	-0.55	-0.79	-0.68	-0.33	NA	0.47
*Dermacoccus*	-0.78	-0.52	-0.37	-0.71	-0.22	0.11
*Eggerthella*	0.92	0.84	-0.64	-0.2	0.76	-0.35
*Enterococcus*	-0.83	-0.85	-0.75	-0.77	-0.57	-0.24
*Epulopiscium*	-0.24	-0.52	-0.52	-0.24	-0.22	-0.22
*Eubacterium*	0.83	0.72	-0.85	-0.47	0.62	-0.35
*Facklamia*	-0.78	-0.77	-0.41	-0.64	-0.03	0.47
*Faecalibacterium*	0.75	0.36	-0.85	-0.62	0.71	-0.21
*Jeotgalicoccus*	-0.78	-0.83	-0.55	-0.64	-0.02	0.33
*Klebsiella*	-0.85	-0.88	-0.86	-0.84	-0.18	0.15
*Kocuria*	-0.68	-0.52	-0.42	-0.62	-0.12	0.16
*Lactobacillus*	0.06	-0.3	0.85	0.83	-0.61	0.25
*Nocardiopsis*	-0.85	-0.79	-0.64	-0.81	NA	0.41
*Oceanobacillus*	-0.75	-0.23	-0.23	-0.75	NA	NA
*Pediococcus*	-0.34	-0.73	-0.28	0.21	-0.55	-0.05
*Propionibacterium*	NA	-0.31	-0.21	0.2	-0.12	0.05
*Proteus*	-0.85	-0.76	-0.77	-0.85	-0.21	-0.23
*Pseudomonas*	-0.5	-0.88	-0.88	-0.5	-0.12	-0.12
*Ruminococcus*	0.83	0.69	-0.85	-0.52	0.67	-0.22
*Salinicoccus*	-0.8	-0.75	-0.45	-0.37	-0.21	0.32
*Serratia*	-0.55	-0.67	-0.67	-0.55	-0.22	-0.22
*Staphylococcus*	-0.84	-0.85	-0.66	-0.68	-0.26	0.31
*Streptomyces*	-0.6	-0.88	-0.87	-0.56	-0.18	-0.03
*Trichococcus*	-0.78	-0.73	-0.56	-0.7	-0.22	0.25
Unclassified *Actinobacteria*	-0.85	-0.86	-0.68	-0.8	-0.37	-0.06
Unclassified *Aerococcaceae*	-0.55	-0.61	-0.58	-0.51	NA	0.2
Unclassified *Bacilli*	-0.79	-0.64	-0.33	-0.54	-0.20	0.25
Unclassified *Catabacteriaceae*	0.92	0.53	-0.79	-0.2	0.87	-0.29
Unclassified *Clostridiaceae*	-0.08	-0.48	-0.25	0.15	-0.29	-0.09
Unclassified *Clostridiales*	0.79	0.6	-0.85	-0.71	0.74	-0.16
Unclassified *Dermabacteraceae*	-0.8	-0.79	-0.62	-0.72	-0.29	0.11
Unclassified *Dietziaceae*	-0.65	-0.67	-0.54	-0.53	0.05	0.38
Unclassified *Enterobacteriaceae*	-0.72	-0.75	-0.85	-0.84	0.18	-0.57
Unclassified *Enterococcaceae*	-0.84	-0.86	-0.49	-0.71	-0.65	-0.03
Unclassified *Erysipelotrichaceae*	0.72	0.11	-0.82	-0.48	0.62	-0.38
Unclassified *Lachnospiraceae*	0.83	0.83	-0.85	-0.3	0.73	-0.22
Unclassified *Lactobacillales*	-0.84	-0.85	-0.58	-0.7	-0.58	-0.06
Unclassified *Planococcaceae*	0.52	0.03	-0.76	-0.24	0.61	-0.12
Unclassified *RF39*	0.84	0.49	-0.83	-0.45	0.80	-0.29
Unclassified *Rickettsiales*	-0.53	-0.88	-0.81	-0.01	-0.43	0.23
Unclassified *Ruminococcaceae*	0.83	0.72	-0.85	-0.72	0.81	-0.32
Unclassified *Streptophyta*	-0.6	-0.88	-0.74	0.17	-0.51	0.54
*Vagococcus*	-0.85	-0.76	-0.67	-0.84	NA	0.29
*Weissella*	-0.81	-0.74	-0.41	-0.46	-0.30	0.32
*Yaniella*	-0.58	-0.31	-0.14	-0.51	-0.12	0.15

Positive correlated taxa are coloured red, while negatively associated taxa are coloured yellow and grey indicates non-significant association. Significance was set a P<0.05 level. NA indicates that a particular genus was absent in both sample types.

## Discussion

In this study, the microbial community structures of non-invasive samples (dust, excreta and litter) were different from invasive samples (caecal and ileal contents), particularly on the abundance of the detected OTUs. However, the majority of OTUs detected in the caecal and ileal contents were also detected in non-invasive samples. This suggests that dust, litter or excreta could be used to determine the presence/absence of most of OTUs present in invasive samples. Similarly, the microbial community structure of non-invasive samples was also different between sample types but the majority of OTUs were detected in all non-invasive sample types.

The results partially support the proposition that compared to invasive samples, the microbial communities of non-invasive samples will show similarities in the detected OTUs but quantitative differences in abundance of the OTUs. Although the majority of the OTUs detected in invasive samples were also detected in non-invasive samples, a higher number of unique OTUs were detected in dust, excreta and litter compared to caecal and ileal contents as reflected in differences in beta-diversity measures. The microbiota of the gut is primarily composed of anaerobic bacteria [60] whereas litter and dust samples contain both aerobic bacteria and the nucleic acid remnants of obligate anaerobes, similarly to excreta. This could be a reason for the higher number of OTUs in dust, excreta and litter compared to ileal and caecal contents. When comparing the number of OTUs that were shared between different sample types, 98–100% OTUs detected in caecal contents and 95–99.8% of OTUs detected in ileal contents were present in non-invasive samples, suggesting that non-invasive samples could be used to identify presence and absence of most of the OTUs of invasive samples. The majority of OTUs detected in ileal or caecal contents that were not detected in non-invasive samples were of low abundance. This is similar to [61], which demonstrated that ~99% of the OTUs found in caecal samples were present in excreta and ~87% of the OTUs found in ileal samples were present in excreta, but higher than [62], reporting that ~60% of the OTUs found in caecal swabs were present in the cloacal swabs. Differences among studies could be attributed due to differences in sample size, sampling techniques, age of the birds, DNA extraction methods, feed, breed or other factors.

In this study, abundance of bacterial phylum *Firmicutes* in caecal contents and dust, caecal contents and litter, ileal contents and dust, and ileal contents and litter were strongly and positively correlated while the abundance of the phylum *Tenericutes* in caecal contents and excreta, and caecal contents and litter were strongly positively correlated. This indicates that, under the conditions of this study, dust and litter could be used as a proxy for ileal and caecal contents to study the abundance of the phylum *Firmicutes*, and excreta as a proxy for caecal contents to study the abundance of the phylum *Tenericutes*. Similarly, litter could also be used as a proxy for caecal contents to study the abundance of phylum *Tenericutes* but not necessarily to investigate the abundance of specific OTUs. None of the non-invasive sample types could be used as a proxy to map the overall microbial composition of invasive samples. Similar to our findings, previous research has shown that arbitrarily collected excreta and cloacal swabs cannot be used as a reference to accurately monitor the microbiota of caecum or ileum [12, 63]. Contrarily, when caecal droppings were sampled, there was a correlation between the microbiota of this sample type and the caecum microbiota [12, 31].

The results partially support the second proposition that the comparison of microbial communities of non-invasive samples will show similarities in the detected OTUs and their abundance. A higher number of unique OTUs was detected in the dust compared to excreta and litter, as depicted by beta diversity measures. The difference in substrate requirements for growth and multiplication of different bacterial species [64] may influence species distribution and abundance level of the same bacterium in different sample types. When comparing the number of OTUs shared between dust and excreta, and litter and dust, 99.2% of the total OTUs found in excreta and litter were detected in the dust. The OTUs exclusive to excreta and litter had low abundance.

The similarity of OTUs shared between non-invasive samples highlights health concerns as it is well-known that aerosolised poultry dust and litter can pose serious issues for farm workers and birds [65]. Culture of settled poultry dust collected from meat chickens and laying hen farms has found an average total bacteria of 3.2 × 10^9^ colony forming units (cfu)/g dust, and actinomycetes at 5.5 × 10^6^ cfu/g, and fungi at 1.2 × 10^6^ cfu/g [65]. Commonly cultivated bacteria in settled dust were *Bacillus*, *Clostridia*, *Corynebacterium*, *Enterobacter*, *Flavobacterium*, *Pseudomonas*, *Staphylococcus* and *E*. *coli*; in addition several biological toxins, allergens and odorous gases have been also detected [65]. Likewise, poultry litter can harbour high levels of culturable potential pathogens such as *Salmonella* and *Campylobacter* [66] in addition to antibiotic resistance genes that may persist in horticultural soils amended with poultry litter [67]. A previous study using a culture-based methodology has shown a differential capacity of aerosolization and survival of bacteria present in litter samples of four commercial meat chicken farms during summer and winter grow-outs, which was affected by the environment temperature and relative humidity [66]. Although *Campylobacter* was detected at high levels in poultry litter (10^7^ most probable number [mpn]/g), it could not be isolated from aerosols inside the poultry house, while *Salmonella* (10^3^–10^5^ mpn/g) could be cultured from aerosols and settled dust and *E*. *coli* was readily isolated in litter and aerosols (10^2^–10^5^ cfu/g). It appears, however, that the survival of these microorganisms outside of tunnel-ventilated meat chicken houses is limited to 20 m beyond the exhaust fan [68].

In this study, no difference in the microbial community structure between challenged and unchallenged groups was observed. The challenge of birds induced subclinical necrotic enteritis as previously described [45] and the lack of difference is unexpected as several studies have reported marked differences in the microbiota of challenged birds in similar experiments [69–71]. A possible explanation for this is the small sample size of the current study which reduced the power to detect changes between groups. In the current study, 18–20 samples were collected for each sample type after inoculation with *Eimeria* spp and *C*. *perfringens* or sham-inoculation (5 sampling times, 2–4 samples per sampling time). Although the use of 8–10 samples per treatment (challenged or control group) per sample type would provide sufficient power for differentiation of the microbiota between treatments [69–71], combining the samples from birds of different ages is likely to have increased the variation in the microbiota of the chickens. Additionally, the microbial composition from samples collected at later stages, i.e., at d 35 is unlikely to differ between treatments as the birds recovered from subclinical necrotic enteritis soon after challenge. All these factors may have contributed to the lack of difference between treatments. A larger sample size should be used and combination of samples from different age should be avoided in future studies to reduce issues of individual bird variation in microbiota studies. Functional analysis of the microbiota would provide additional insights into the capabilities of the microbial communities of different sample types.

In conclusion, dust, excreta, and litter could not be used to map the overall microbial composition of caecal and ileal contents; however, they could be used to detect the presence and absence of the majority of the OTUs found in caecal and ileal contents. This could potentially be useful for screening poultry flocks for bacterial taxa of interest such as pathogenic bacteria or for investigating the effects of microbial feed additives using specific molecular assays or changes in microbial community structure after management interventions. This study provides insight on the use of non-invasive sample types to monitor intestinal microbiota. Further investigation on a larger sample size would be useful to determine the relationship between the microbiota of diverse sample types and their relationship with gut health and production parameters.

## Supporting information

S1 Table Ingredients and nutrient composition of industry-standard diet of grower (days 7–21) and finisher (days 21–35) [18]. (DOCX)

S2 Table Difference in the microbial community between challenged and unchallenged groups measured via permutational multivariate analysis of variance (PERMANOVA) on Bray–Curtis and Jaccard distance metric using the Adonis function. PERMDISP = permutational multivariate dispersions test calculated using Bray-Curtis and Jaccard distance metrics. R-square is the proportion of the variance explained by the group. Cumulative data from days 13–35 were used (n = 92 samples). The challenged group received 2,500 sporulated oocyst of *E*. *brunetti*, and 5,000 sporulated oocysts of *E*. *maxima* and *E*. *acervulina* on day 9 and 10^8^ colony forming units of *C*. *perfringens* on days 14 and 15. The unchallenged group received 1 ml sterile phosphate buffer solution on day 9 and sterile broth on day 14 and 15.
(DOCX)

S3 Table Total number of operational taxonomic units (OTUs) found in invasive and non-invasive samples. (XLSX)

S4 Table Operational taxonomic units (OTUs) that are shared between two or more sample types. (XLSX)

S5 Table Operational taxonomic units (OTUs) that are found in one sample type but absent in another. (XLSX)

S6 Table Overall spearman’s rank correlation coefficient (R) between invasive and non-invasive samples at OTU level. (DOCX)

S7 Table Spearman’s rank correlation coefficient (R) between different samples at phylum level. (DOCX)

S1 Fig Hierarchical clustering of the different sample types (ileal and caecal contents, excreta, litter and dust) using A) Bray–Curtis and B) Jaccard distance metrics. (DOCX)
