# Supplementary material for: Microbial communities of poultry house dust, excreta and litter are partially representative of microbiota of chicken caecum and ileum
Source: PLoS One. 2021 Aug 5;16(8):e0255633. doi: 10.1371/journal.pone.0255633 (PMC8341621; doi:10.1371/journal.pone.0255633)
Supplement: S1 Table — (DOCX) [file pone.0255633.s001.docx]

# **S1 Table.** Ingredients and nutrient composition of industry-standard diet of grower (days 7–21) and finisher (days 21- 35) [18].

| **Ingredients (%)** | **Grower (7-21)** | **Finisher (21-35)** |
| --- | --- | --- |
| Wheat (10.4% CP) | 49.67 | 54.24 |
| Soybean meal (46.2% CP) | 31.25 | 26.38 |
| Sorghum (11.1% CP) | 12.45 | 13.60 |
| Canola oil | 3.42 | 2.97 |
| Alpha cellulose | 0.00 | 0.00 |
| Dicalcium phosphate | 0.479 | 0.285 |
| Limestone | 0.984 | 0.933 |
| Sodium chloride | 0.177 | 0.177 |
| Sodium bicarbonate | 0.150 | 0.150 |
| Potassium carbonate | 0.000 | 0.000 |
| Xylanase^1^ | 0.005 | 0.005 |
| Phytase^2^ | 0.010 | 0.010 |
| Titanium dioxide | 0.500 | 0.500 |
| Vitamin premix^3^ | 0.060 | 0.050 |
| Mineral premix^4^ | 0.100 | 0.100 |
| L-Lys SO_4_ | 0.282 | 0.247 |
| D, L-Met | 0.315 | 0.261 |
| L-Thr | 0.092 | 0.078 |
| L-Val | 0.030 | 0.008 |
| Gly | 0.000 | 0.000 |
| L-Ile | 0.000 | 0.000 |
| L-Arg | 0.000 | 0.000 |
| L-Leu | 0.000 | 0.000 |
| L-His | 0.000 | 0.000 |
| L-Phe | 0.000 | 0.000 |
| L-Trp | 0.000 | 0.000 |
| Choline chloride | 0.025 | 0.020 |
| Nutrients |  |  |
| AMEn, kcal/kg | 3,080 | 3,100 |
| CP | 21.6 | 19.8 |
| SID^5^ Met | 0.573 | 0.504 |
| SID TSAA | 0.840 | 0.760 |
| SID Lys | 1.130 | 1.000 |
| SID Thr | 0.720 | 0.650 |
| SID Val | 0.890 | 0.800 |
| SID Gly | 0.727 | 0.668 |
| SID Gly_equiv_ | 1.619 | 1.489 |
| SID Ile | 0.780 | 0.710 |
| SID Arg | 1.239 | 1.111 |
| SID Leu | 1.410 | 1.301 |
| SID His | 0.473 | 0.432 |
| SID Phe | 0.916 | 0.838 |
| SID Phe + Tyr | 1.589 | 1.452 |
| SID Trp | 0.245 | 0.225 |
| Calcium | 0.750 | 0.680 |
| Available Phosphorus | 0.380 | 0.340 |
| Sodium | 0.177 | 0.177 |
| Potassium | 0.963 | 0.881 |
| Chloride | 0.160 | 0.160 |
| Choline | 1,600 | 1,500 |
| Linoleic acid | 1.757 | 1.657 |
| DEB^6^ mEq/kg | 279 | 258 |

**Abbreviations:** AA- amino acids; AMEn- apparent metabolizable energy corrected for nitrogen; Arg-arginine; CP-crude protein; DEB-dietary electrolyte balance; EAA-essential amino acids; Gly_equiv_- glycine equivalence; His-histidine; Leu-leucine; Ile-isoleucine; Lys-lysine; Met-methionine; Phe-phenylalanine; SP-standard protein; Thr-threonine; Trp-tryptophan; TSAA-total sulfur amino acids; Tyr-tyrosine; Val-valine.

^1^Econase XT 25 (AB Vista, 1000 BXU/kg). ^2^ Quantum Blue, 5 G (AB Vista, 500 FTU/kg). ^3^Vitamin premix per kg diet: vitamin A-12 MIU; vitamin D-5 MIU; vitamin E-75 mg; vitamin K-3 mg; nicotinic acid-55 mg; pantothenic acid-13 mg; folic acid-2 mg; riboflavin-8 mg; cyanocobalamin-0.016 mg; biotin-0.25 mg; pyridoxine-5 mg; thiamine-3 mg; antioxidant-50 mg. ^4^Mineral premix per kg diet: Cu-16 mg as copper sulfate; Mn-60 mg as manganese sulfate; Mn-60 mg as manganous oxide; I-0.125 mg as potassium iodide; Se-0.3 mg; Fe-40 mg as iron sulfate; Zn-50 mg as zinc oxide; Zn-50 mg as zinc sulfate. ^5^Digestible coefficients for raw ingredients determined using AMINODat 5.0 (Evonik Animal Nutrition). ^6^DEB mEq/kg calculated as 10,000 × (Na^+^ K^+^- Cl^-^).
