# Supplementary material for: Microbial communities of poultry house dust, excreta and litter are partially representative of microbiota of chicken caecum and ileum
Source: PLoS One. 2021 Aug 5;16(8):e0255633. doi: 10.1371/journal.pone.0255633 (PMC8341621; doi:10.1371/journal.pone.0255633)
Supplement: S2 Table — PERMDISP = permutational multivariate dispersions test calculated using Bray-Curtis and Jaccard distance metrics. R-square is the proportion of the variance explained by the group. Cumulative data from days 13–35 were used (n = 92 samples). The challenged group received 2,500 sporulated oocyst of E. brunetti, and 5,000 sporulated oocysts of E. maxima and E. acervulina on day 9 and 108 colony forming units of C. perfringens on days 14 and 15. The unchallenged group received 1 ml sterile phosphate buffer solution on day 9 and sterile broth on day 14 and 15. (DOCX) [file pone.0255633.s002.docx]

# **S2 Table**. Difference in the microbial community between challenged and unchallenged groups measured via permutational multivariate analysis of variance (PERMANOVA) on Bray–Curtis and Jaccard distance metric using the Adonis function. PERMDISP = permutational multivariate dispersions test calculated using Bray-Curtis and Jaccard distance metrics. R-square is the proportion of the variance explained by the group. Cumulative data from days 13-35 were used (n = 92 samples). The challenged group received 2,500 sporulated oocyst of *E. brunetti,* and 5,000 sporulated oocysts of *E. maxima* and *E. acervulina* on day 9 and 10^8^ colony forming units of *C. perfringens* on days 14 and 15. The unchallenged group received 1 ml sterile phosphate buffer solution on day 9 and sterile broth on day 14 and 15.

|  | **Challenged vs unchallenged group** | | | | | | |
| --- | --- | --- | --- | --- | --- | --- | --- |
|  | Adonis Bray-Curtis | | |  | Adonis Jaccard | | |
|  | R^2^ | P-value | PERMDISP P value |  | R^2^ | P-va P-value | PERMDISP  P value |
| Caecal contents | 0.08 | 0.26 | 0.75 |  | 0.08 | 0.24 | 0.75 |
| Dust | 0.006 | 0.99 | 0.66 |  | 0.01 | 0.99 | 0.67 |
| Excreta | 0.02 | 0.83 | **0.02** |  | 0.03 | 0.77 | **0.02** |
| Ileal contents | 0.07 | 0.31 | 0.87 |  | 0.07 | 0.37 | 0.85 |
| Litter | 0.11 | 0.07 | 0.30 |  | 0.10 | 0.06 | 0.34 |
