# Supplementary material for: Microbial communities of poultry house dust, excreta and litter are partially representative of microbiota of chicken caecum and ileum
Source: PLoS One. 2021 Aug 5;16(8):e0255633. doi: 10.1371/journal.pone.0255633 (PMC8341621; doi:10.1371/journal.pone.0255633)
Supplement: S6 Table — (DOCX) [file pone.0255633.s006.docx]

# **S6 Table.** Overall spearman’s rank correlation coefficient (R) between invasive and non-invasive samples at OTU level

| **Comparison** | **Spearman’s rank correlation coefficient (R)** | **P-value** |
| --- | --- | --- |
| Dust and Caecum | 0.41 | <0.001 |
| Excreta and Caecum | 0.58 | <0.001 |
| Ileum and Dust | 0.34 | <0.001 |
| Ileum and Excreta | 0.49 | <0.001 |
| Litter and Caecum | -0.46 | <0.001 |
| Litter and Ileum | 0.55 | <0.001 |
