# Supplementary material for: Microbial communities of poultry house dust, excreta and litter are partially representative of microbiota of chicken caecum and ileum
Source: PLoS One. 2021 Aug 5;16(8):e0255633. doi: 10.1371/journal.pone.0255633 (PMC8341621; doi:10.1371/journal.pone.0255633)
Supplement: S7 Table — (DOCX) [file pone.0255633.s007.docx]

# **S7 Table.** Spearman’s rank correlation coefficient (R) between different samples at phylum level

|  | **Caecal contents and dust** | | **Caecal contents and litter** | | **Ileal contents and dust** | | **Ileal contents and litter** | | **Ileal contents and excreta** | | **Caecal contents and excreta** | |
| --- | --- | --- | --- | --- | --- | --- | --- | --- | --- | --- | --- | --- |
| **Taxa** | P-value | R | P-value | R | P-value | R | P-value | R | P-value | R | P-value | R |
| *Actinobacteria* | **<0.001** | -0.71 | **<0.001** | -0.82 | **<0.001** | -0.50 | **<0.001** | -0.71 | 0.29 | 0.17 | 0.31 | 0.16 |
| *Firmicutes* | **<0.001** | 0.85 | **<0.001** | 0.83 | **<0.001** | 0.75 | **<0.001** | 0.77 | 0.10 | 0.002 | 0.07 | -0.28 |
| *Proteobacteria* | **<0.001** | -0.80 | **<0.001** | -0.78 | **<0.001** | -0.85 | **<0.001** | -0.83 | **0.01** | -0.39 | 0.56 | 0.09 |
| *Tenericutes* | **<0.001** | 0.60 | **<0.001** | 0.81 | **<0.001** | -0.85 | **<0.001** | -0.68 | **0.04** | -0.32 | **<0.001** | 0.78 |
