# Supplementary material for: Microbial communities of poultry house dust, excreta and litter are partially representative of microbiota of chicken caecum and ileum
Source: PLoS One. 2021 Aug 5;16(8):e0255633. doi: 10.1371/journal.pone.0255633 (PMC8341621; doi:10.1371/journal.pone.0255633)
Supplement: S1 Fig — (DOCX) [file pone.0255633.s008.docx]

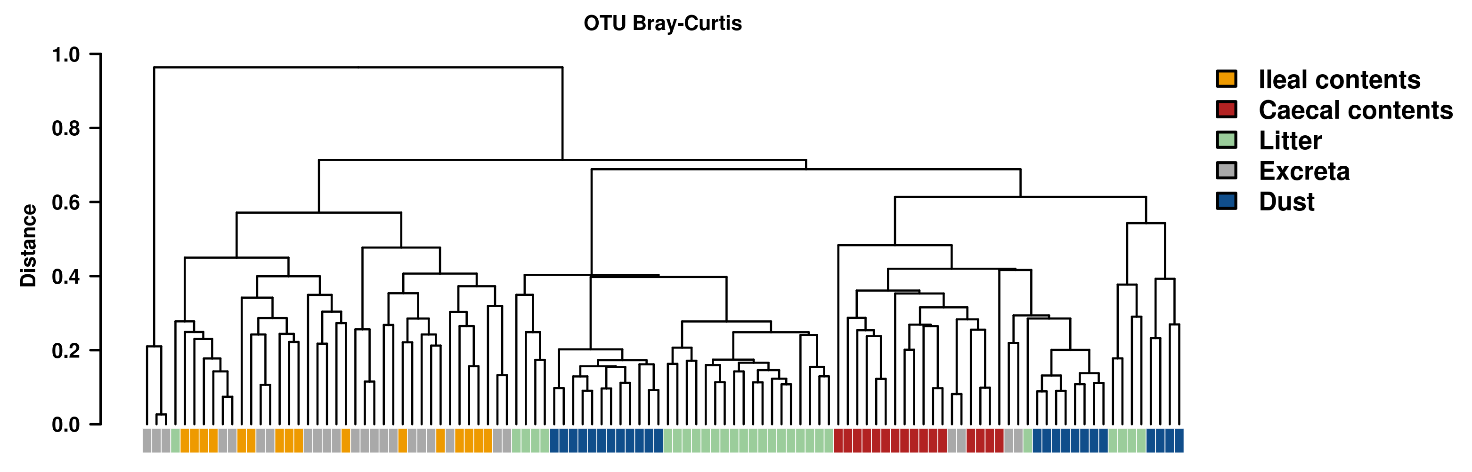

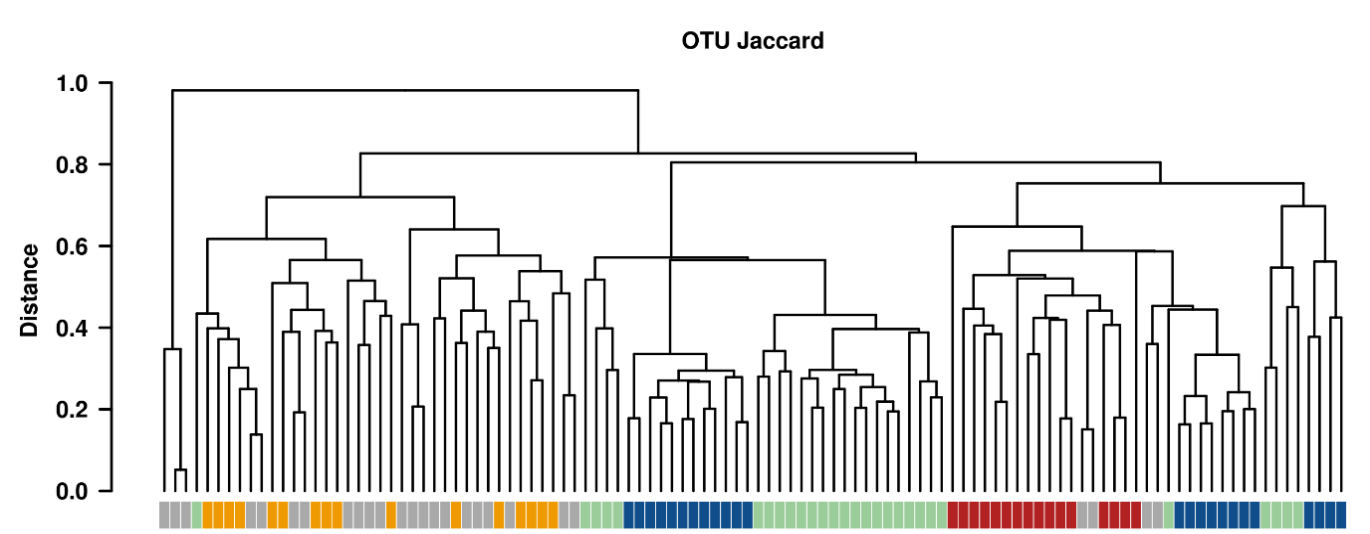

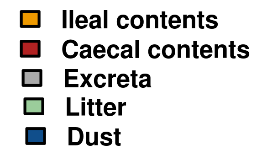


**B)**

**Sample types**

**S1 Fig.** Hierarchical clustering of the different samples (ileal and caecal contents, excreta, litter and dust) using A) Bray─Curtis and B) Jaccard distance metrics.
